# Comparison of ziprasidone and olanzapine on cognitive function in patients with schizophrenia at different stages: a prospective study in Huai’an, China

**DOI:** 10.3389/fnbeh.2025.1561615

**Published:** 2025-10-24

**Authors:** Jingjing Yao, Zeliang Liu, Xiaoming Xiao, Liu Zhang, Shouhui Qi, Yangcheng Ge, Hua Han, Xiuling Wang

**Affiliations:** ^1^Department of Psychiatry, Jinhu People’s Hospital, Huai'an, China; ^2^Department of Imaging, Jinhu People’s Hospital, Huai'an, China

**Keywords:** schizophrenia, antipsychotic drugs, drug combination, cognitive function, ziprasidone

## Abstract

**Objective:**

To compare the effects of ziprasidone and olanzapine on cognitive function in patients with first-episode schizophrenia and chronic schizophrenia at different stages.

**Methods:**

Cognitive function tests were performed on chronic schizophrenic patients who took olanzapine for a long time, first-episode drug-free schizophrenic patients, and healthy controls.

**Results:**

There were significant differences in the digit span test, Stroop color and word test, auditory verbal learning test N2, N3, N4, trail-making test, verbal fluency test, and clock drawing test between first-episode drug-free schizophrenic patients and healthy controls (*p* < 0.05). Compared with patients with chronic schizophrenia, there were significant differences in the digit span test, Stroop color and word test B, auditory verbal learning test, trail making test B, and clock drawing test in patients with first-episode schizophrenia after 4 weeks of olanzapine treatment (*p* < 0.05). Compared with patients with chronic schizophrenia after 4 weeks of Ziprasidone treatment, patients with first-episode schizophrenia had significant differences in the digit span test, Stroop color, and word test, auditory verbal learning test N3, and clock drawing test after 4 weeks of olanzapine treatment (*p* < 0.05). Compared with patients with chronic schizophrenia who were treated with Ziprasidone for 12 weeks, there were significant differences in Stroop color and word test A, auditory verbal learning test N3, and clock drawing test in patients with first-episode schizophrenia after 4 weeks of olanzapine treatment (*p* < 0.05).

**Conclusion:**

Patients with schizophrenia have cognitive dysfunction in the early stage of onset. The combination of ziprasidone and olanzapine can effectively improve cognitive dysfunction and promote the recovery of social functions of patients.

## 1 Introduction

Schizophrenia is a severe mental disorder with the main clinical manifestations of dysfunctions in thinking, emotion, and behavior, as well as uncoordinated mental activities and significantly decreased social function (Carpentier et al., 2025). Patients with schizophrenia have a wide range of cognitive dysfunction, which often exists independently before the onset of the disease (Cao et al., 2022). With the prolongation of the course of the disease, the degree of cognitive impairment gradually increases (Yu et al., 2024). The more extensive the impact on the social functions of learning, work, and marriage, the greater the economic and mental burden on the patient’s family and society (Shimada et al., 2022).

Atypical antipsychotics are often selected by healthcare professionals based on patients’ clinical characteristics, including prior treatment response, tolerability profile, comorbidities (e.g., cardiovascular risk 2), and guideline recommendations. After systematic treatment, most patients can restore their social functions (Petric et al., 2024). Atypical antipsychotics may improve cognitive function through dopamine receptor modulation (e.g., antagonism or partial agonism), as reflected in cortical circuit activity (Torrisi et al., 2020). Additionally, antipsychotics (e.g., atypical agents) may reduce N-methyl-D-aspartate (NMDA) receptor activity, thereby disrupting the function of the cortico-limbic-thalamic circuit and subsequently modulating cognitive performance (Krzystanek and Pałasz, 2019). Other mechanisms include alterations in catecholaminergic neuronal activity, which diminish the pro-cognitive enhancer effects on cortical circuits (Minzenberg et al., 2018), as well as functional impacts on brain regions (e.g., neuroplastic changes in areas associated with cognitive control) (Cadena et al., 2018). It has been reported that atypical antipsychotic drugs can improve the cognitive function of patients with schizophrenia (Wang and Qiu, 2013; Luo et al., 2004).

Among them, olanzapine can improve clinical symptoms and cognitive function by blocking the secretion pathway of 5-hydroxytryptamine and dopamine and inhibiting the pathway of dopamine in the brain. However, olanzapine has no significant effect on the improvement of attention, learning ability, and memory (Liu, 2018). At present, several studies have shown that ziprasidone can improve cognitive dysfunction (Teng et al., 2020; Wang et al., 2019; Huang et al., 2023). Ziprasidone can not only improve the positive and negative symptoms but also improve the cognitive function of patients with schizophrenia (Zhang, 2017). It can promote the recovery of social function, help patients return to society, and reduce the family’s economic burden. As one of the most commonly used antipsychotics worldwide, the comparison between olanzapine and newer drugs such as ziprasidone can provide practical references for clinical decision-making. Therefore, this study aimed to explore the effects of olanzapine and ziprasidone on cognitive function, to provide some ideas for clinicians in drug use, and to provide a certain direction for patients with schizophrenia to improve their cognitive function.

## 2 Subjects and methods

### 2.1 Subjects

This study was approved by the Jinhu County People’s Hospital Ethical Committee (No. LLSC2021–82). All subjects signed the informed consent. From March 2021 to June 2023, 18 patients with chronic schizophrenia who had long-term use of olanzapine and 34 first-episode drug-free schizophrenic patients who were hospitalized in the Psychiatric Department of Jinhu County People’s Hospital were included in this study. Psychiatrists screened all patients at the attending and above levels using the International Classification of Diseases (ICD-10) to confirm the diagnosis.

Inclusion criteria for chronic schizophrenia: (1) patients with a course of disease ≥ 2 years (mean disease duration: 9.4 ± 5.1 years), and long-term use of olanzapine; (2) right-handed; (3) aged over 18 years old and under 60 years old. Exclusion criteria for chronic schizophrenia: (1) patients with a history of head trauma; (2) nervous system diseases; (3) mental retardation; (4) a history of substance dependence in the past six months; (5) pregnant and lactating women; (6) undergoing electroconvulsive therapy.

Inclusion criteria for first-episode drug-free schizophrenic patients: (1) patients with the first onset, the course of disease less than two years (mean disease duration: 14.4 ± 7.2 months), without psychiatric drugs; (2) right-handed; (3) aged over 18 years old and under 60 years old. Exclusion criteria for first-episode drug-free schizophrenic patients: (1) patients with a history of head trauma; (2) nervous system diseases; (3) mental retardation; (4) a history of substance dependence in the past six months; (5) pregnant and lactating women; (6) undergoing electroconvulsive therapy.

From March 2021 to June 2023, 29 healthy volunteers from the family members of the staff, the staff of the canteen, the staff of training, and the assistant training of Jinhu County People’s Hospital affiliated to Yangzhou University were recruited. They are all right-handed. Each healthy control was screened by a professional psychiatrist using the Structured Clinical Interview for DSM-IV axis I disorders, non-patient version (SCID-I/NP) to exclude a history of mental disorders.

### 2.2 Data collection and scale assessment

On the first day of enrollment, a professional psychiatrist completed the collection of demographic and clinical data of first-episode drug-free schizophrenic patients and patients with chronic schizophrenia, including disease duration and medication history. All participants completed cognitive function tests, including a digit span test, a semantic similarity test, a Stroop color and word test, an auditory verbal learning test, a trail-making test, a verbal fluency test, and a clock drawing test.

First-episode drug-free schizophrenic patients were treated with olanzapine within one week after admission, titrated up to a sufficient amount of 20 mg/day. After 4 weeks, cognitive function tests were performed. Patients with chronic schizophrenia, who had been on long-term olanzapine therapy (mean disease duration: 9.4 ± 5.1 years), were switched to ziprasidone, titrated to 120 mg/day within 1 week. The patients were tested for cognitive function after 4 weeks and 12 weeks of treatment. Equivalent dose conversions were considered to ensure comparability between treatment groups. Long-term use of olanzapine was defined as continuous administration exceeding 52 weeks.

Except for the healthy control group, the two patient groups were assessed after drug intervention using the Brief Psychiatric Rating Scale (BPRS) to gauge symptom severity and the Treatment Emergent Symptom Scale (TESS) to monitor adverse effects (Supplementary Figure 1).

### 2.3 Digit span test

The digit span test is used to evaluate working memory capacity, including forward recall (repeating digit sequences) and backward recall (reciting in reverse order), reflecting the frontal–parietal circuit’s function. The test was conducted in a quiet environment. The examiner read numerical sequences at a rate of one digit per second, starting with 2–3 digits and progressively increasing by one digit per level. Each sequence length was administered at least twice. For the forward digit span (FDS) task, subjects were required to immediately repeat the numbers in the same order to assess short-term memory. For the backward digit span (BDS) task, subjects were instructed to recall the digits in reverse order to evaluate working memory. Scoring could be performed using either partial credit (awarding points for correctly positioned digits) or traditional scoring (only fully correct sequences received points). The maximum correct sequence length and raw scores were recorded and compared with normative data to determine percentile ranks or standardized scores. The test duration was approximately 5–10 min, and background noise was minimized. Results should be interpreted in conjunction with other cognitive assessment tools. Testing was discontinued if the subject failed two consecutive trials or met the predefined discontinuation criteria.

### 2.4 Semantic similarity test

Semantic similarity tests require subjects to generalize abstract relationships between word pairs (e.g., “apple-banana” in the fruit category). During the test design phase, semantic categories (e.g., disease symptoms) were defined based on specific ontologies (e.g., HPO/UMLS), and structured word pairs (e.g., “apple-banana” representing “fruit”) were constructed while controlling lexical variables and integrating multi-source evidence. During implementation, word pairs were presented to participants via a computerized interface, requiring them to perform similarity scoring (0–1) or category selection tasks (e.g., determining whether the words belonged to the same category, such as “fruit”), enabling real-time data collection and potential adaptation to individual differences. Similarity computation employed multivariate models: distributed semantic representations were learned using vector space models (e.g., word embeddings), or ontology-based methods were applied to calculate path distances between concepts. For processing large-scale clinical data, parallelized frameworks (e.g., MapReduce) were utilized to efficiently extract features, employing specific metrics (e.g., Relative Best Pair, dual similarity mechanisms). For result evaluation, reliability was validated by comparing human ratings (e.g., Pearson correlation), and the scores were applied to classification/ranking tasks (e.g., disease identification), assessment of neural functional associations (e.g., temporal lobe/default mode network), or clustering multiple scores in cohort studies to generate interpretable phenotypic class descriptions.

### 2.5 Stroop color and word test

The Stroop Color-Word Test measures selective attention, inhibitory control, and cognitive flexibility through a “color-naming conflict” paradigm. The test consists of three parts: the word condition, which requires participants to quickly read out the names of printed color words (e.g., the word “red” printed in black font); the color condition, where participants must swiftly identify the color of the printed ink (e.g., the word “blue” printed in red ink); and the color-word condition, which challenges participants to suppress the semantic interference of the word and only name the ink color. Completion time and error rates reflect frontal lobe cortex function and are highly correlated with impairments in executive function.

### 2.6 Auditory verbal learning test

The Auditory Verbal Learning Test (AVLT) assesses immediate and delayed recall, recognition, and forgetting rates through multiple rounds of word list learning, reflecting the function of the hippocampus-medial temporal lobe memory system. The examiner orally presented a 15-word list (List A) at a rate of 1 word per second. After each trial, the subject was required to perform immediate free recall. This procedure was repeated for 5 trials, with the learning slope calculated as the difference between trial 5 and trial 1 scores. Subsequently, an interference word list (List B) was introduced to evaluate proactive inhibition. After a 5-min delay, unprompted recall of List A was assessed (short-delay recall), followed by another recall test after 20–30 min (long-delay recall). The forgetting rate was calculated as [(short-delay score - long-delay score)/short-delay score] × 100%. During the recognition phase, a mixed list containing words from List A, List B, and novel words (30–40 words total) was presented. Memory discrimination was analyzed using: (1) Discriminability indices (d’/A’); (2) Response bias measures (B″); (3) Dual-process recognition paradigms to distinguish between true and false memories. Advanced analyses included: (1) Semantic clustering index (LBC) to evaluate organizational strategies; (2) Serial position effect analysis to detect primacy/recency abnormalities; (3) Learning curve modeling to predict medial temporal lobe function (flattened slopes suggesting encoding impairment).

### 2.7 Trail making test

The Trail Making Test (TMT) consists of two parts: Part A, which requires connecting numbers in sequential order, and Part B, which involves alternately connecting numbers and letters. This test assesses attentional switching, visual search speed, and executive functions. Abnormalities in Part B are associated with frontal lobe damage. TMT-A: Participants sequentially connect randomly arranged numbered dots (typically 25 dots) in ascending order using a pen or touch-based tool, drawing continuous straight lines without lifting the device while avoiding numerical omissions or sequence errors. This task measures visual scanning, visuomotor speed, and basic attention, with performance scored by completion time (in seconds). TMT-B: Participants alternately connect numbers and letters in an interleaved sequence. Under equivalent speed and accuracy requirements, this task additionally assesses divided attention, attentional switching, and cognitive flexibility. Errors and completion time are recorded. The primary outcome measure is the time taken to complete each part (timed from the first to the last dot). Derived metrics include TMT B-A (the time difference between TMT-B and TMT-A) and TMT B/A (the time ratio between TMT-B and TMT-A), which isolate executive functions (e.g., set-shifting and mental flexibility) by controlling for baseline visuomotor skills. Prolonged TMT-B completion time or increased errors may indicate frontal lobe dysfunction, demonstrating sensitivity to visual search and attentional switching deficits. A computerized version can be implemented to capture detailed movement trajectories.

### 2.8 Verbal fluency test

The Verbal Fluency Test comprises phonemic fluency (such as listing words beginning with “F” within 1 min) and semantic fluency (such as listing words within the “animal” category), which reflect the left frontal lobe’s language generation ability and semantic network integration capability. The test must be conducted in a standardized environment. Specific phonemic letters (e.g., F, A, S—determined through pilot studies to optimize difficulty levels and linguistic applicability) or semantic categories (e.g., animals or common objects) should be selected. During administration, the examiner provides clear instructions in a quiet room, requiring the participant to generate non-repetitive words as quickly and accurately as possible within a fixed duration (typically 60 s). The examiner must time the task and record responses using audio devices or written transcripts to ensure complete data capture. Post-assessment, the total number of correct words should be documented. Additionally, clustering (e.g., consecutive production of semantically related words) and switching strategies (e.g., transitions between distinct categories) must be analyzed to provide deeper insights into cognitive processes. Scoring should account for potential errors, such as rule violations or repeated responses, using systematic guidelines to ensure consistent evaluation. Throughout the process, variables including age, education level, and gender must be controlled. Where applicable, culturally adapted normative data should be referenced to enhance the test’s validity and reliability.

### 2.9 Clock drawing test

The Clock Drawing Test is utilized to evaluate executive function, visuospatial abilities, and planning skills. Participants were instructed to independently complete the task on an A4 sheet of paper following standardized verbal commands: Draw a clock face with numbers 1–12, set the hands to 11:10—the long hand pointing to 2 and the short hand near 11. The drawing sequence (contour → number placement → hand positioning) was observed. The digital version (dCDT) allowed for the recording of dynamic details (pauses/erasures/drawing speed) in response to planning ability. Abnormal features included: Visuospatial deficits: Distorted clock face, lateral clustering of numbers, disproportionate spacing. Executive dysfunction: Omitted/misordered numbers, incorrect hand placement (e.g., failure to depict 11:10). Impaired planning: Lack of strategy, frequent erasures, boundary violations. Scoring followed the Rouleau 10-point system: Clock structure (closed contour/completeness of numbers/positioning/spacing; max 4 points). Hand accuracy (differentiation of long/short hands/correct time; max 6 points). The Shulman system demonstrated higher clinical sensitivity. Clinical correlations: Frontal lobe deficits led to command neglect or conceptual errors. Parietal lobe lesions caused left-sided neglect or clock face tilting (e.g., number crowding common in Parkinson’s disease). The dCDT enhanced detection of mild cognitive impairment (AUC = 0.83) via: Temporal analysis (>180 s). Pressure-sensitive digitization (tremulous lines indicating motor dysfunction).

### 2.10 Statistical analysis

Statistical analysis was performed using SPSS 26.0 statistical software. The age, years of education, and cognitive function of 34 first-episode drug-free schizophrenic patients were compared with those of 18 patients with chronic schizophrenia who had long-term use of olanzapine and 29 healthy controls by Analysis of Variance (ANOVA) and independent sample *t*-test. The chi-square test was used to compare genders. The age of first onset, years of education, and cognitive function tests were compared between 18 patients with chronic schizophrenia who had long-term use of olanzapine and 15 first-episode drug-free schizophrenic patients who were treated with olanzapine for 4 weeks (some patients dropped out due to early discharge or poor compliance) by independent sample *t*-test. Gender was also compared by the chi-square test. The age of first onset, years of education, and cognitive function tests were compared among 15 first-episode drug-free schizophrenic patients who were treated with olanzapine for 4 weeks, patients with chronic schizophrenia who had long-term use of olanzapine and treated with ziprasidone for 4 weeks (15 cases, some patients dropped out due to early discharge or poor compliance) and 12 weeks (13 cases, some patients dropped out due to self-withdrawal) by ANOVA and independent sample *t*-test. Gender was also compared by the Chi-square test. Paired-sample t-tests and/or ANOVA were used for within-group comparisons. Detailed study procedures and statistical methods are shown in Supplementary Figure 1. *p* < 0.05 was considered statistically significant.

## 3 Results

### 3.1 Comparison of schizophrenic patients with healthy controls

The results showed significant differences among the three groups in years of education. On cognitive function tests, except for the Trail-Making Test A, Trail-Making Test B, and Clock Drawing Test, all other indicators showed statistically significant differences (Supplementary Table 1). There were no statistically significant differences in disease duration and medication dosage between groups (Supplementary Table 1), suggesting that these factors did not confound the cognitive outcomes.

### 3.2 Comparison of first-episode drug-free schizophrenic patients with healthy controls

There were no significant differences in age, gender, and education level between first-episode drug-free schizophrenic patients and healthy controls (*p* > 0.05), and the effects of age, gender, and education level on cognitive function were excluded (Table 1).

**Table 1:** General demographic data statistics between patients and healthy controls.

Items	First-episode drug-free schizophrenic patients (*n* = 31)	Healthy controls (*n* = 29)	*p*
Age (years)	23.81 ± 5.46	22.10 ± 3.53	0.17
Gender (male/female)	21/10	16/13	0.43
Education level (years)	12.19 ± 3.11	13.90 ± 2.78	0.068

The scores of digit span test (*p* = 0.003), semantic similarity test (*p* = 0.00), auditory verbal learning test N1 (*p* = 0.00), N2 (*p* = 0.00), N3 (*p* = 0.00), N4 (*p* = 0.00), verbal fluency test (*p* = 0.00) and clock drawing test (*p* = 0.024) in first-episode drug-free schizophrenic patients were significantly lower than those in healthy controls. The time spent on Stroop color and word test A (*p* = 0.00), B (*p* = 0.00), trail-making test A (*p* = 0.00), and B (*p* = 0.00) in first-episode drug-free schizophrenic patients was significantly higher than that of healthy controls (Table 2).

**Table 2:** Comparison of cognitive function between first-episode drug-free schizophrenic patients and healthy controls.

Items		First-episode drug-free schizophrenic patients (*n* = 31)	Healthy controls (*n* = 29)	*p*
Digit span test	Total	13.07 ± 2.32	15.17 ± 2.84	0.003^**^
Semantic similarity test		15.52 ± 5.28	20.86 ± 3.40	0.00^**^
Stroop color and word test A	Time	26.36 ± 9.63	18.86 ± 3.16	0.00^**^
Stroop color and word test B	Time	48.45 ± 19.21	30.72 ± 6.69	0.00^**^
Auditory verbal learning test	N1	3.03 ± 1.11	5.24 ± 1.62	0.00^**^
	N2	5.10 ± 1.80	7.93 ± 2.07	0.00^**^
	N3	6.39 ± 2.03	9.41 ± 1.82	0.00^**^
	N4	4.26 ± 2.46	8.35 ± 2.19	0.00^**^
Trail-making test A	Time	62.55 ± 19.73	38.93 ± 8.98	0.00^**^
Trail-making test B	Time	128.16 ± 35.25	75.52 ± 15.22	0.00^**^
Verbal fluency test		19.48 ± 5.70	26.90 ± 5.86	0.00^**^
Clock drawing test		8.48 ± 1.75	9.31 ± 0.89	0.024^*^

*p < 0.05; **p < 0.01.

### 3.3 Comparison of cognitive function between first-episode drug-free schizophrenic patients and patients with chronic schizophrenia who had long-term use of olanzapine

There were no significant differences in age of first onset, gender, and education level between first-episode drug-free schizophrenic patients and patients with chronic schizophrenia who had long-term use of olanzapine (*p* > 0.05), and the effects of age of first onset, gender, and education level on cognitive function were excluded (Table 3).

**Table 3:** General demographic data statistics between first-episode drug-free schizophrenic patients and patients with chronic schizophrenia who had long-term use of olanzapine.

Items	First-episode drug-free schizophrenic patients (*n* = 33)	Patients with chronic schizophrenia (*n* = 18)	*p*
Age (years)	23.55 ± 5.83	26.72 ± 9.16	0.20
Gender (male/female)	22/11	13/5	0.76
Education level (years)	11.85 ± 3.18	10.00 ± 3.09	0.051

The scores of the digit span test (*p* = 0.026), semantic similarity test (*p* = 0.004), auditory verbal learning test N2 (*p* = 0.013), N3 (*p* = 0.023), N4 (*p* = 0.002), and clock drawing test (*p* = 0.00) in first-episode drug-free schizophrenic patients were significantly higher than those in patients with chronic schizophrenia who had long-term use of olanzapine. The time spent on Stroop color and word test A (*p* = 0.014), B (*p* = 0.013), trail-making test A (*p* = 0.023), and B (*p* = 0.007) in first-episode drug-free schizophrenic patients were significantly higher than those in patients with chronic schizophrenia who had long-term use of olanzapine (Table 4).

**Table 4:** Comparison of cognitive function between first-episode drug-free schizophrenic patients and patients with chronic schizophrenia who had long-term use of olanzapine.

Items		First-episode drug-free schizophrenic patients (*n* = 33)	Patients with chronic schizophrenia (*n* = 18)	*p*
Digit span test	Total	12.73 ± 1.97	10.78 ± 3.17	0.026^*^
Semantic similarity test		14.94 ± 5.64	12.39 ± 5.67	0.13
Stroop color and word test A	Time	26.88 ± 9.26	36.85 ± 14.44	0.014^*^
Stroop color and word test B	Time	50.09 ± 18.52	71.03 ± 30.12	0.013^*^
Auditory verbal learning test	N1	2.91 ± 1.10	2.44 ± 1.65	0.24
	N2	4.94 ± 1.73	3.61 ± 1.79	0.013^*^
	N3	6.15 ± 1.91	4.72 ± 2.37	0.023^*^
	N4	4.03 ± 2.44	1.83 ± 1.98	0.002^**^
Trail-making test A	Time	62.82 ± 19.24	109.78 ± 79.25	0.023^*^
Trail-making test B	Time	133.82 ± 41.04	261.03 ± 173.95	0.007^**^
Verbal fluency test		19.00 ± 5.57	13.72 ± 6.83	0.004^**^
Clock drawing test		8.49 ± 1.70	5.67 ± 2.72	0.00^**^

*p < 0.05; **p < 0.01.

### 3.4 Comparison of cognitive function between first-episode drug-free schizophrenic patients who took olanzapine for 4 weeks and patients with chronic schizophrenia who had long-term use of olanzapine

There were no significant differences in age of first onset, gender, and education level between first-episode drug-free schizophrenic patients who took olanzapine for 4 weeks and patients with chronic schizophrenia who had long-term use of olanzapine (*p* > 0.05, Table 5).

**Table 5:** General demographic data statistics between first-episode drug-free schizophrenic patients who took olanzapine for 4 weeks and patients with chronic schizophrenia who had long-term use of olanzapine.

Items	First-episode drug-free schizophrenic patients (*n* = 15)	Patients with chronic schizophrenia (*n* = 15)	*p*
Age (years)	21.53 ± 4.12	25.20 ± 8.94	0.17
Gender (male/female)	12/3	12/3	1.00
Education level (years)	12.60 ± 2.78	10.80 ± 2.73	0.084

The scores of digit span test (*p* = 0.006), auditory verbal learning test N1 (*p* = 0.008), N2 (*p* = 0.032), N3 (*p* = 0.014), N4 (*p* = 0.014) and clock drawing test (*p* = 0.00) in first-episode drug-free schizophrenic patients who taken olanzapine for 4 weeks were significantly higher than those in patients with chronic schizophrenia who had long-term use of olanzapine. The time spent on Stroop color and word test B (*p* = 0.021) and trail making test B (*p* = 0.032) in first-episode drug-free schizophrenic patients who took olanzapine for 4 weeks was significantly lower than that in patients with chronic schizophrenia who had long-term use of olanzapine (Table 6).

**Table 6:** Comparison of cognitive function between first-episode drug-free schizophrenic patients who took olanzapine for 4 weeks and patients with chronic schizophrenia who had long-term use of olanzapine.

Items		First-episode drug-free schizophrenic patients (*n* = 15)	Patients with chronic schizophrenia (*n* = 15)	*p*
Digit span test	Total	14.13 ± 2.10	11.13 ± 3.31	0.006^*^
Semantic similarity test		17.13 ± 3.54	13.53 ± 5.40	0.04^*^
Stroop color and word test A	Time	25.13 ± 5.18	33.89 ± 13.66	0.028^*^
Stroop color and word test B	Time	46.33 ± 12.82	69.20 ± 32.60	0.021^*^
Auditory verbal learning test	N1	4.40 ± 1.55	2.73 ± 1.62	0.008^**^
	N2	5.33 ± 1.76	3.87 ± 1.81	0.032^*^
	N3	7.33 ± 2.26	5.13 ± 2.33	0.014^*^
	N4	4.40 ± 2.64	2.13 ± 2.03	0.014^*^
Trail-making test A	Time	54.20 ± 17.64	94.72 ± 74.70	0.058
Trail-making test B	Time	125.27 ± 41.36	224.87 ± 159.05	0.032^*^
Verbal fluency test		23.40 ± 30.68	15.07 ± 6.49	0.31
Clock drawing test		9.33 ± 0.90	6.00 ± 2.45	0.00^**^

*p < 0.05; **p < 0.01.

### 3.5 Comparison of cognitive function between first-episode drug-free schizophrenic patients who had taken olanzapine for 4 weeks and patients with chronic schizophrenia who had long-term use of olanzapine and taken ziprasidone for 4 weeks

The scores of the digit span test (*p* = 0.023), auditory verbal learning test N3 (*p* = 0.015), and clock drawing test (*p* = 0.00) in first-episode drug-free schizophrenic patients who had taken olanzapine for 4 weeks were significantly higher than those in patients with chronic schizophrenia who had long-term use of olanzapine and taken ziprasidone for 4 weeks. The time spent on Stroop color and word test A (*p* = 0.04) and Stroop color and word test B (*p* = 0.045) in first-episode drug-free schizophrenic patients who had taken olanzapine for 4 weeks were significantly lower than those in patients with chronic schizophrenia who had long-term use of olanzapine and taken ziprasidone for 4 weeks (Table 7). No statistically significant difference in BPRS outcomes was observed between the two groups after 4 weeks of treatment. The incidence of adverse events was also similar between the groups; all events were mild elevations in liver enzymes, with a TESS score of 1 (Supplementary Table 2).

**Table 7:** Comparison of cognitive function between first-episode drug-free schizophrenic patients who had taken olanzapine for 4 weeks and patients with chronic schizophrenia who had long-term use of olanzapine and taken ziprasidone for 4 weeks.

Items		First-episode drug-free schizophrenic patients (*n* = 15)	Patients with chronic schizophrenia (*n* = 15)	*p*
Digit span test	Total	14.13 ± 2.10	11.53 ± 3.60	0.023^*^
Semantic similarity test		17.13 ± 3.54	17.47 ± 4.81	0.83
Stroop color and word test A	Time	25.13 ± 5.18	35.12 ± 16.59	0.04^*^
Stroop color and word test B	Time	46.33 ± 12.82	62.40 ± 26.10	0.045^*^
Auditory verbal learning test	N1	4.40 ± 1.55	3.40 ± 1.64	0.097
	N2	5.33 ± 1.76	4.67 ± 1.91	0.33
	N3	7.33 ± 2.26	5.20 ± 2.24	0.015^*^
	N4	4.40 ± 2.64	3.20 ± 2.73	0.23
Trail-making test A	Time	54.20 ± 17.64	81.89 ± 54.57	0.079
Trail-making test B	Time	125.27 ± 41.36	207.66 ± 145.04	0.05
Verbal fluency test		23.40 ± 30.68	17.20 ± 6.42	0.45
Clock drawing test		9.33 ± 0.90	6.60 ± 2.35	0.001^**^

*p < 0.05; **p < 0.01.

### 3.6 Comparison of cognitive function between first-episode drug-free schizophrenic patients who had taken olanzapine for 4 weeks and patients with chronic schizophrenia who had long-term use of olanzapine and taken ziprasidone for 12 weeks

There were no significant differences in age of first onset, gender, and education level between first-episode drug-free schizophrenic patients who had taken olanzapine for 4 weeks and patients with chronic schizophrenia who had long-term use of olanzapine and taken ziprasidone for 12 weeks (*p* > 0.05, Table 8).

**Table 8:** General demographic data statistics between first-episode drug-free schizophrenic patients who had taken olanzapine for 4 weeks and patients with chronic schizophrenia who had long-term use of olanzapine and taken ziprasidone for 12 weeks.

Items	First-episode drug-free schizophrenic patients (*n* = 15)	Patients with chronic schizophrenia (*n* = 15)	*p*
Age (years)	21.53 ± 4.12	23.31 ± 7.22	0.44
Gender (male/female)	12/3	10/3	1.00
Education level (years)	12.60 ± 2.78	10.85 ± 2.88	0.11

The time spent on Stroop color and word test A (*p* = 0.036) in first-episode drug-free schizophrenic patients who had taken olanzapine for 4 weeks was significantly lower than that of patients with chronic schizophrenia who had long-term use of olanzapine and taken ziprasidone for 12 weeks (Table 9). The auditory verbal learning test N3 (*p* = 0.025) and clock drawing test (*p* = 0.003) in first-episode drug-free schizophrenic patients who took olanzapine for 4 weeks were significantly higher than those in patients with chronic schizophrenia who had long-term use of olanzapine and took ziprasidone for 12 weeks (Table 9).

**Table 9:** Comparison of cognitive function between first-episode drug-free schizophrenic patients who had taken olanzapine for 4 weeks and patients with chronic schizophrenia who had long-term use of olanzapine and taken ziprasidone for 12 weeks.

Items		First-episode drug-free schizophrenic patients (*n* = 15)	Patients with chronic schizophrenia (*n* = 15)	*p*
Digit span test	Total	14.13 ± 2.10	12.31 ± 3.07	0.074
Semantic similarity test		17.13 ± 3.54	18.08 ± 5.82	0.62
Stroop color and word test A	Time	25.13 ± 5.18	30.20 ± 6.94	0.036^*^
Stroop color and word test B	Time	46.33 ± 12.82	65.78 ± 31.04	0.52
Auditory verbal learning test	N1	4.40 ± 1.55	3.77 ± 1.83	0.33
	N2	5.33 ± 1.76	4.62 ± 2.18	0.34
	N3	7.33 ± 2.26	5.46 ± 1.85	0.025^*^
	N4	4.40 ± 2.64	3.92 ± 2.43	0.63
Trail-making test A	Time	54.20 ± 17.64	115.62 ± 136.03	0.13
Trail-making test B	Time	125.27 ± 41.36	198.39 ± 172.40	0.16
Verbal fluency test		23.40 ± 30.68	19.85 ± 7.87	0.69
Clock drawing test		9.33 ± 0.90	7.23 ± 1.96	0.003^**^

*p < 0.05; **p < 0.01.

### 3.7 Comparison within schizophrenic patients before and after treatment

Among patients with chronic schizophrenia, BPRS scores and performance on the Semantic Similarity Test and Auditory Verbal Learning Test-N4 showed statistically significant differences at baseline, 4 weeks, and 12 weeks (Supplementary Table 3). Among first-episode schizophrenia patients, BPRS scores and scores on the Auditory Verbal Learning Test-N1 and Clock Drawing Test differed significantly between baseline and 4 weeks (Supplementary Table 4).

## 4 Discussion

Digital span tests and verbal fluency tests are widely used cognitive function assessment tools, which can reflect the impairment of abstract generalization ability, concept formation, cognitive transfer, memory, attention, and speed (Bogers et al., 2024). Auditory verbal learning test mainly reflects working and verbal memory (immediate and delayed memory). The semantic similarity test reflects the ability to perform abstract generalization. The clock drawing test examines a more comprehensive cognitive function. The Stroop color and word test examines the information processing speed, such as sorting function, attention, and mental activity ability.

In this study, we found that the scores of the digit span test, semantic similarity test, auditory verbal learning test, verbal fluency test, and clock drawing test in the first-episode drug-free schizophrenic patients were lower than those in the healthy controls. In contrast, the time spent in the Stroop color and word test and the trail making test was higher than that in healthy controls, indicating that the patients’ abstract generalization ability, memory, attention, and information processing speed were abnormal. It suggests that the first-episode schizophrenic patients had extensive cognitive dysfunction in the early stage of the disease. It has been reported that cognitive dysfunction is an independent symptom of schizophrenia. At least 80% of patients with schizophrenia have persistent and severe cognitive impairment, especially in attention, verbal memory, and executive function (Raffard, 2023). Our results are consistent with most studies.

In this study, for the first-episode drug-free schizophrenic patients and the first-episode patients who took olanzapine for 4 weeks, their scores in the digit span test, auditory verbal learning test, verbal fluency test, and clock drawing test were higher than those of chronic schizophrenia patients who took olanzapine for a long time, while the Stroop color and word test and the trail making test took less time than those of chronic schizophrenia patients who took olanzapine for a long time, suggesting that with the extension of the course of the disease, even in the case of taking the same antipsychotic drugs, the cognitive dysfunction of chronic schizophrenia patients continues to increase, which may be related to hospitalization time, course of disease, frequency of onset, medication compliance and other factors. Secondly, the pharmacological mechanism of olanzapine has no significant effect on the improvement of attention, learning ability, and memory in patients with schizophrenia (Teng et al., 2020). Therefore, olanzapine may not be as effective as ziprasidone in improving cognitive function.

In this study, we found that after 4 weeks of olanzapine treatment, the digit span test score, recall number of verbal learning test N3, and clock drawing test score of first-episode schizophrenic patients were still higher than those of chronic schizophrenia patients treated with olanzapine combined with ziprasidone, while the time spent on Stroop color and word teat was lower than that of chronic schizophrenia patients treated with olanzapine combined with ziprasidone. In addition, there was no significant difference between the two groups in the scores of the auditory word learning test, verbal fluency test, and trail-making test. It shows that although the cognitive function of patients with chronic schizophrenia after treatment with olanzapine combined with ziprasidone is still worse than that of first-episode schizophrenic patients, some cognitive functions, such as executive function, attention, and mental activity, are constantly improving. The recall number of auditory word learning test N3 and the score of clock drawing test of first-episode schizophrenic patients after 4 weeks of olanzapine treatment were higher than those of patients with chronic schizophrenia treated by olanzapine combined with ziprasidone for 12 weeks, while the time spent on Stroop color and word test was lower than that of patients with chronic schizophrenia treated by olanzapine combined with ziprasidone for 12 weeks. It shows that the memory, attention, and speed of patients with chronic schizophrenia are also recovering. Therefore, the longer the combination of ziprasidone, the more comprehensive the recovery of cognitive function in patients with chronic schizophrenia, and the more they return to society and promote the recovery of social function. Studies have found that patients who need to be treated with other antipsychotic drugs may show improvements in cognitive function after the use of ziprasidone (Bogers et al., 2024). Long-term use of ziprasidone can gradually restore the cognitive function of patients with chronic schizophrenia to normal (Bogers et al., 2024), and also promote advanced cognitive function (Zhong et al., 2024), and maybe a long-term change (Lungu et al., 2024). Taken together, long-term use of ziprasidone can improve the quality of life of patients with chronic schizophrenia, continuously improve their treatment compliance, increase their confidence in treatment, and help them gradually return to society.

Several studies have shown that ziprasidone is comparable to olanzapine in the control of negative and positive symptoms in patients with schizophrenia (Kelebie et al., 2025), and can effectively control cognitive dysfunction (Zhang et al., 2021), and should not be less prone to adverse drug reactions such as glucose and lipid metabolism and weight (Wang et al., 2024). Therefore, whether patients have first-episode or chronic schizophrenia, clinicians can prefer atypical antipsychotic drugs in the choice of antipsychotic drugs, especially drugs that antagonize the uptake of 5-hydroxytryptamine and norepinephrine. They can comprehensively improve the patient’s mental symptoms and cognitive function, improve the cognition of the disease and medication compliance, reduce the disease recurrence rate, help patients return to society, increase self-confidence, and reduce the economic and mental burden of the family and society. It also provides some ideas for clinicians to choose the diagnosis and treatment plan, and pay attention to improving cognitive dysfunction.

However, the effects of antipsychotic drugs on cognitive function exhibit complexity and remain controversial. Atypical antipsychotics (e.g., aripiprazole, quetiapine), due to their 5-HT₂ receptor antagonism, induce fewer extrapyramidal side effects compared to first-generation agents and may potentially improve cognitive performance. The dopaminergic mechanism constitutes the core pathway through which antipsychotics enhance cognition, primarily by modulating prefrontal cortex function to influence working memory and executive function, though substantial interindividual variability in response exists (Torrisi et al., 2020). Long-term antipsychotic use demonstrates a nonlinear association with cognitive changes. Dose-reduction strategies (e.g., olanzapine or risperidone tapering) may yield cognitive benefits but require careful risk–benefit assessment regarding relapse (Singh et al., 2022; Collin, 2024), whereas discontinuation could preserve specific cognitive domains such as semantic fluency and processing speed (Collin, 2024). Antipsychotics should not be regarded as cognitive enhancers, as their modest cognitive benefits likely stem from the alleviation of positive symptoms rather than direct procognitive effects (Feber et al., 2024). Notably, drug-induced metabolic disorders (e.g., obesity, dyslipidemia) may exacerbate cognitive impairment. Taken together, future research should focus on drug repositioning and personalized treatment strategies to address the heterogeneity in cognitive responses.

This study adopted a prospective design to compare patients at different stages of the disease, incorporating a healthy control group for the first time, to systematically evaluate differences in multiple cognitive dimensions (such as the Stroop test and verbal learning), providing evidence for stage-specific treatments. The study also verified the impact of drug intervention time windows (4 weeks and 12 weeks) on cognitive improvement, which has clinical guidance significance. However, there are some limitations to this study. Firstly, the sample size is small and confined to a single region. Secondly, variations in dosage and the effects of concomitant medications were not controlled. The evaluation period was relatively short (up to 12 weeks), lacking long-term follow-up.

## Data Availability

The raw data supporting the conclusions of this article will be made available by the authors, without undue reservation.
