# Supplementary material for: Comparison of ziprasidone and olanzapine on cognitive function in patients with schizophrenia at different stages: a prospective study in Huai’an, China
Source: Front Behav Neurosci. 2025 Oct 24;19:1561615. doi: 10.3389/fnbeh.2025.1561615 (PMC12592029; doi:10.3389/fnbeh.2025.1561615)
Supplement: Supplementary file 1 [file Data_Sheet_1.DOCX]

**Supplementary files**


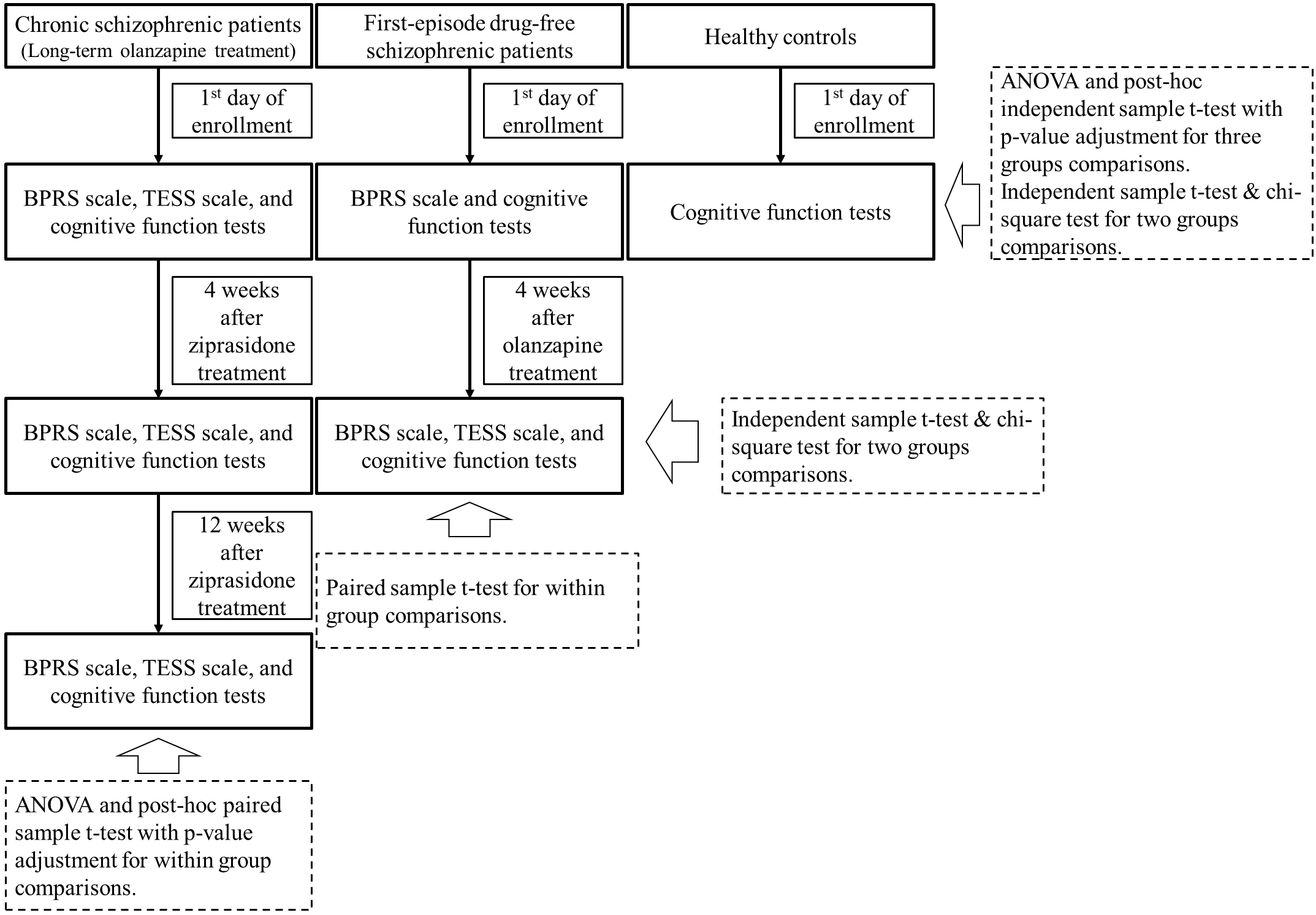


**Supplementary figure 1. Study workflow and statistical methods.**

**Supplementary table 1. Comparison of baseline characteristic data among three groups of participants**

| **Items** | **First-episode drug-free schizophrenic patients (n=33)** | **Patients with chronic schizophrenia (n=18)** | **Healthy controls (n=29)** | **Chi2/F** | **p value** |
| --- | --- | --- | --- | --- | --- |
| Age (years) | 23.55 ± 5.83 | 26.72 ± 9.16 | 22.10 ± 3.53 | 0.517 | 0.474 |
| **Disease duration**(years) | **1.2 ± 0.6** | **9.4 ± 5.1** | – | – | **n.s.** |
| Gender (male/female) | 22/11 | 13/5 | 16/13 | 1.708 | 0.426 |
| Education level (years) | 11.85 ± 3.18 | 10.00 ± 3.09 | 13.90 ± 2.78 | 4.683 | 0.033 |
| **Medication dosage (mg/day)** | **14.56 ± 5.02** | **15.16 ± 4.65** | **–** | **–** | **n.s.** |
| Digit span test (Total) | 13.07 ± 2.32 | 10.78 ± 3.17 | 15.17 ± 2.84 | 7.656 | 0.007 |
| Semantic similarity test | 15.52 ± 5.28 | 12.39 ± 5.67 | 20.86 ± 3.40 | 16.124 | <0.001 |
| Stroop color and word test A (Time) | 26.36 ± 9.63 | 36.85 ± 14.44 | 18.86 ± 3.16 | 6.765 | 0.011 |
| Stroop color and word test B (Time) | 48.45 ± 19.21 | 71.03 ± 30.12 | 30.72 ± 6.69 | 9.216 | 0.003 |
| Auditory verbal learning test N1 | 3.03 ± 1.11 | 2.44 ± 1.65 | 5.24 ± 1.62 | 30.089 | <0.001 |
| Auditory verbal learning test N2 | 5.10 ± 1.80 | 3.61 ± 1.79 | 7.93 ± 2.07 | 25.552 | <0.001 |
| Auditory verbal learning test N3 | 6.39 ± 2.03 | 4.72 ± 2.37 | 9.41 ± 1.82 | 25.12 | <0.001 |
| Auditory verbal learning test N4 | 4.26 ± 2.46 | 1.83 ± 1.98 | 8.35 ± 2.19 | 30.586 | <0.001 |
| Trail-making test A (Time) | 62.55 ± 19.73 | 109.78 ± 79.25 | 38.93 ± 8.98 | 3.465 | 0.066 |
| Trail-making test B (Time) | 128.16 ± 35.25 | 261.03 ± 173.95 | 75.52 ± 15.22 | 3.951 | 0.05 |
| Verbal fluency test | 19.48 ± 5.70 | 13.72 ± 6.83 | 26.90 ± 5.86 | 17.12 | <0.001 |
| Clock drawing test | 8.48 ± 1.75 | 5.67 ± 2.72 | 9.31 ± 0.89 | 1.404 | 0.24 |

**Supplementary table 2. Comparison of BPRS and TESS results before and after treatment in first-episode and chronic patients**

| Items | First-episode drug-free schizophrenic patients (n=33) | Patients with chronic schizophrenia (n=18) | t/chi2 | p value |
| --- | --- | --- | --- | --- |
| BPRS on enrollment | 62.21±2.89 | 62.39±2.59 | -0.233 | 0.817 |
| BPRS after 4 weeks treatment | 40.24±2.43 | 40.33±2.74 | -0.127 | 0.899 |
| TESS after 4 weeks treatment 0 score | 26 | 13 |  |  |
| TESS after 4 weeks treatment 1 score | 7 | 5 | 0.279 | 0.597 |

**Supplementary table 3. Comparison of patients with chronic schizophrenia at enrollment, after 4 weeks and 12 weeks of treatment**

| Items | | Enrollment (n=18) | 4 weeks after ziprasidone treatment(n=18) | 12 weeks after ziprasidone treatment(n=15) | F | p value |
| --- | --- | --- | --- | --- | --- | --- |
| BPRS |  | 62.39±2.59 | 40.33±2.74 | 36.44±2.01(n = 18) | 242.171 | <0.001 |
| Digit span test | Total | 10.78±3.17 | 11±3.5 | 12±2.95 | 1.146 | 0.29 |
| Semantic similarity test |  | 12.39±5.67 | 16±5.83 | 17.27±5.91 | 6.056 | 0.017 |
| Stroop color and word test A | Time | 36.85±14.44 | 38.33±16.88 | 30.04±6.5 | 1.83 | 0.182 |
| Stroop color and word test B | Time | 71.03±30.12 | 64.56±24.5 | 69.54±32.01 | 0.034 | 0.853 |
| Auditory verbal learning test | N1 | 2.44±1.65 | 3.11±1.64 | 3.53±1.81 | 3.502 | 0.067 |
|  | N2 | 3.61±1.79 | 4.33±1.91 | 4.47±2.07 | 1.737 | 0.194 |
|  | N3 | 4.72±2.37 | 4.89±2.19 | 5±2.24 | 0.127 | 0.723 |
|  | N4 | 1.83±1.98 | 2.83±2.62 | 3.67±2.47 | 5.065 | 0.029 |
| Trail-making test A | Time | 109.78±79.25 | 96±62.63 | 125.37±128.58 | 0.198 | 0.659 |
| Trail-making test B | Time | 261.03±173.95 | 241.61±158.39 | 227.95±182.23 | 0.317 | 0.576 |
| Verbal fluency test |  | 13.72±6.83 | 15.78±6.9 | 18.2±8.5 | 3.066 | 0.086 |
| Clock drawing test |  | 5.67±2.72 | 6.39±2.35 | 7.07±2.02 | 2.849 | 0.098 |

**Supplementary table 4. Comparison of outcomes in first-episode schizophrenic patients at enrollment and after 4 weeks of treatment**

| Items |  | Enrollment (n = 33) | 4 weeks after olanzapine treatment (n = 15) | t | p value |
| --- | --- | --- | --- | --- | --- |
| BPRS |  | 62.21±2.89 | 40.24±2.43 (n = 33) | 33.958 | <0.001 |
| Digit span test | Total | 12.94±2.31 | 14.13±2.1 | -1.776 | 0.086 |
| Semantic similarity test |  | 15.12±5.65 | 17.13±3.54 | -1.513 | 0.138 |
| Stroop color and word test A | Time | 26.56±9.3 | 25.5±5.22 | 0.514 | 0.61 |
| Stroop color and word test B | Time | 49.41±18.66 | 46.33±12.82 | 0.669 | 0.508 |
| Auditory verbal learning test | N1 | 2.97±1.14 | 4.4±1.55 | -3.21 | 0.004 |
|  | N2 | 5±1.74 | 5.33±1.76 | -0.613 | 0.545 |
|  | N3 | 6.26±1.99 | 7.33±2.26 | -1.582 | 0.127 |
|  | N4 | 4.12±2.46 | 4.4±2.64 | -0.352 | 0.728 |
| Trail-making test A | Time | 62.79±18.94 | 54.2±17.64 | 1.536 | 0.135 |
| Trail-making test B | Time | 135.09±41.08 | 125.27±41.36 | 0.768 | 0.449 |
| Verbal fluency test |  | 19.15±5.55 | 23.4±30.68 | -0.533 | 0.602 |
| Clock drawing test |  | 8.53±1.69 | 9.33±0.9 | -2.163 | 0.036 |
